# UC2 search: using unique connectivity of uncharged compounds for metabolite annotation by database searching in mass spectrometry-based metabolomics

**DOI:** 10.1093/bioinformatics/btx649

**Published:** 2017-10-12

**Authors:** Nozomu Sakurai, Takafumi Narise, Joon-Soo Sim, Chang-Muk Lee, Chiaki Ikeda, Nayumi Akimoto, Shigehiko Kanaya

**Affiliations:** 1 Department of Technology Development, Kazusa DNA Research Institute, Kisarazu, Chiba, Japan; 2 Department of Agricultural Biotechnology, National Institute of Agricultural Sciences, Jeonju, Korea; 3 Graduate School of Information Science, Nara Institute of Science and Technology, 8916-5 Takayama, Ikoma, Nara, Japan

## Abstract

**Summary:**

For metabolite annotation in metabolomics, variations in the registered states of compounds (charged molecules and multiple components, such as salts) and their redundancy among compound databases could be the cause of misannotations and hamper immediate recognition of the uniqueness of metabolites while searching by mass values measured using mass spectrometry. We developed a search system named UC2 (Unique Connectivity of Uncharged Compounds), where compounds are tentatively neutralized into uncharged states and stored on the basis of their unique connectivity of atoms after removing their stereochemical information using the first block in the hash of the IUPAC International Chemical Identifier, by which false-positive hits are remarkably reduced, both charged and uncharged compounds are properly searched in a single query and records having a unique connectivity are compiled in a single search result.

**Availability and implementation:**

The UC2 search tool is available free of charge as a REST web service (http://webs2.kazusa.or.jp/mfsearcher) and a Java-based GUI tool.

**Supplementary information:**

Supplementary data are available at *Bioinformatics* online.

## 1 Introduction

Although a large number of compounds are registered in public databases, the existence of various forms of compounds and their redundancy in compound databases make it difficult to identify or annotate metabolite peaks by mass values that are detected in mass spectrometry (MS)-based metabolomics: (i) some compounds are registered as charged molecules and (ii) some compounds are registered as sets of multiple components such as salts. These two circumstances may cause misannotations when the compounds are searched by their mass values (see Supplementary Results and Discussion 1.1.1, 1.1.2 and 1.1.4). The situation is complicated further because (iii) several variations of stereoisomers can be registered in databases and (iv) the same compound can be registered in several databases. These latter two complications hamper immediate recognition between isomers and redundant records (see Supplementary Results and Discussion 1.1.3). Some issues caused by this state of affairs are partly solved in particular systems. Compounds with the same atomic connectivity are searched in PubChem (Wang *et al.*, 2009) with several combinations of Representational State Transfer Application Programming Interfaces. In ChemSpider (Pence and Williams, 2010), redundancies among the databases are removed, and the entry of salts can be excluded using the filter option of ‘Single/Multi-component’. However, no system exists that resolves all issues caused by the abovementioned four situations, with the first one remaining to be solved. Since there are no direct clues in the MS data to distinguish if the metabolite is detected as an adduct ion (such as [M + H]^+^ or [M − H]^−^) or molecular ion (such as [M]^+^ or [M]^−^ for the positive and negative modes, respectively), searches using neutralized mass values by accounting for the adduct ions and searches using detected *m/z* values are required to obtain hits for both uncharged and charged molecules (such as pigment flavonoids in plants) registered in the databases. This multiple cycle of searches leads to a considerable number of false positives. In this study, 6.3–20.6% of database hits for metabolite peaks from biological samples were estimated as false positives (Table 1). Practical examples of these issues are presented in Supplementary Results and Discussion 1.1.

**Table 1.** The number of the peaks (mass values) searched by the conventional search and the UC2 search

	Tomato^a^		Urine		Random mass values^b^	
	Positive	Negative	Positive	Negative	Positive	Negative
Total	510	359	1264	1475	6491	6379
Results found	277	167	967	1092	1000	1000
In conventional search	277	164	967	1091	998	984
In UC2 search	220	139	906	1012	556	553
Results found only in the conventional search	57 (20.6%)	28 (16.8%)	61 (6.3%)	80 (7.3%)	444 (44.4%)	447 (44.7%)
False positives	57	28	61	80	444	447
Results found only in the UC2 search	0 (0%)	3 (1.8%)	0 (0%)	1 (0.1%)	2 (0.2%)	16 (1.6%)
True positives^c^	0	3	0	1	1	14
False positives	0	0	0	0	1	2

a Metabolites (160 peaks) detected in both positive and negative modes are shown as the positive.

b [M+H]^+^ and [M−H]^-^ were assumed for positive and negative modes, respectively, in the search with randomly generated mass values.

c The queries whose results were found only in UC2 search and matched to charged or fragmented entries were defined as true positives.

To solve all of these issues, we developed a new search system for databases constructed using information of the Unique Connectivity of the atoms of Uncharged Compounds (UC2). The UC2 search system can be the front end of a compound database search for better metabolite annotations in untargeted metabolomics studies.

## 2 Materials and methods

The structural data of compounds were obtained from KEGG (Kanehisa *et al.*, 2016), KNApSAcK (Afendi *et al.*, 2012), a flavonoid database (here referred to as FlavonoidViewer, http://metabolomics.jp/wiki/Category: FL), LIPID MAPS (Fahy *et al.*, 2009), HMDB (Wishart *et al.*, 2013), UNPD (Gu *et al.*, 2013) and PubChem (Wang *et al.*, 2009). The Chemistry Development Kit 2.0 (Willighagen *et al.*, 2017) and Java 7 (Oracle corporation) were used for molecular calculations and generation of the hash of the IUPAC International Chemical Identifier (InChIKey) (Heller *et al.*, 2013). When multiple components are included in a record (hereafter referred to as fragmented records), the one with the largest molecular weight was used as a representative. Positively or negatively charged molecules were tentatively neutralized by removing or adding an equivalent number of hydrogens from or to the formula, respectively (see Supplementary Results and Discussion 1.1.5 for applicability of hydrogen for the adjustment of the charge). While this does not always make chemical sense, it does enable a computationally efficient approach for structural look-up in mass spectral applications. The first block (14 letters) of the standard InChIKey (hereafter referred to as InChIKey skeleton), which shows a unique signature of the same connectivity of atoms, along with tentatively neutralized formula, tentatively neutralized formula weight, original ID and name of the compounds was stored in a database named UC2 using MariaDB. Updates of the data are scheduled per month for PubChem, per week for KEGG and when the databases are updated for the other databases. The web service for searching UC2 was constructed in MFSearcher (Sakurai *et al.*, 2013). The graphical user interface (GUI) tool to search the UC2 database was developed using Java.

For the evaluation of UC2, a list of tomato metabolites was obtained from Iijima *et al.* (2008). Metabolite peaks from human urine were prepared from the raw data (van der Hooft *et al.*, 2016) using an in-house version of PowerGet (Sakurai *et al.*, 2014). For comparison of search results, both a search using UC2 (UC2 search) and a conventional search were performed using the MFSearcher web service and KEGG, KNApSAcK, FlavonoidViewer, LIPID MAPS and HMDB as target databases. For peaks of default adduct ions ([M + H]^+^ and [M − H]^−^ for the positive and negative modes, respectively), the sum of compound records searched with both default adduct ion and detected *m/z* value was used as the candidate number in the conventional search. Details of the methods are provided in the Supplementary Methods.

## 3 Results and discussion

Number of charged entries, unique formulae and unique InChIKey skeletons in the databases are summarized in Supplementary Tables S1–S3. A low ratio of the number of unique formulae to that of unique InChIKey skeletons implies that a considerable number of different constitutional isomers, stereoisomers and fragmented records are registered in the databases. Many charged records were found, particularly in FlavonoidViewer (7.2%). Each database has its own unique InChIKey skeletons, particularly HMDB (only 37% were shared). These results suggest that a search with the proper charge across multiple databases is required to cover the maximum number of compounds and that redundancy of the same compounds among the databases has to be removed. See also Supplementary Results and Discussion 1.3 for details on the features of the database entries.

We developed the UC2 search system to solve these issues. Charged entries are tentatively neutralized by the addition or subtraction of hydrogen to or from the formulae. The tentatively neutralized compounds and neutral compounds were stored in a relational database system with the neutralized mass value and the first block of InChIKey (InChIKey skeleton), which represents connectivity of the atoms. For a record with multiple components, such as a salt, the largest component is used as a representative. The structure of stored data is shown in Supplementary Figure S10. Based on the neutralized mass, both neutral and charged compounds can be searched in a single query (Supplementary Results and Discussion 1.1). Compounds with the same connectivity of atoms searched among the compound databases were compiled in a single result using the InChIKey skeleton. The search functions of UC2 are available on the MFSearcher web service (Sakurai *et al.*, 2013) in a RESTful manner and via a GUI tool provided at the MFSearcher website. The detailed information of the candidate compounds can be retrieved from the original databases. Functions to search compounds by a specified InChIKey skeleton and a formula are also available on the web service. This function can be useful, for example for searching registrations of possible stereoisomers among the databases.

We compared the number of peaks with database hits, number of candidates and appropriateness of the results from a search using UC2 (UC2 search) and a conventional search. A curated metabolite list of tomato and an automatically detected metabolite list of human urine were used as queries. As both neutralized mass values based on the estimated adduct ions and detected *m/z* values were assumed and searched in the conventional search, the results found only in the conventional search or the UC2 search could contain potential false positives. We manually checked these results and found that a considerable number of results were found only in the conventional search (6.3–20.6%) and all of them were false positives (Table 1). Furthermore, most of the false positives were caused by charged entries unexpectedly appearing in the searches with neutralized mass values and detected *m/z* values (Supplementary Table S4, see also Supplementary Results and Discussion 1.1.2 for this case of false positives). A small number of queries gave results only in the UC2 search, containing true positives matched properly to charged and/or fragmented entries, and also false-positive hits for entries with repeat units or mistakes in the structure. For negative modes, there were hits for entries with positive molecular ions. These were considered as true-positive hits because some positive molecular ions, such as anthocyanins, can be detected in the negative mode as [M − 2H]^−^ (Sun *et al.*, 2012). The candidates with unexpected charge in the UC2 results can be detected automatically by comparing the charge of the given adduct ion because the UC2 result contains the signature of the charge in the original database (Supplementary Results and Discussion 1.2). This function is implemented in the MFSearcher GUI tool. These results suggest that a UC2 search remarkably reduces the false positives caused by entries registered as charged compounds. Similar results were also observed when the examination was performed with randomly generated mass values, suggesting that the false positives in the conventional search generally occur independently of the sample; however, the proportion of the number of peaks with search results in the UC2 and the conventional search and the ratio of the number of queries with unique results were sample dependent. The cause might be that the mass values of mathematically possible molecules are not uniformly distributed (Kind and Fiehn, 2006).

More than half of the peaks (67–81%) showed a smaller number of candidates in the UC2 results (Supplementary Figs S12–S14, Supplementary Table S4). In the UC2 results, a larger number of peaks (17–34%) had only one candidate. This suggests that removal of redundancy among the databases by InChIKey skeletons together with removal of the false positives mentioned above contributes to getting more concise and interpretable results for annotating metabolite peaks.

The UC2 search system is a computationally efficient approach to deal with database redundancy, and it could help improve the annotation of metabolites in untargeted metabolomics. Although none of the approaches developed so far, including the UC2 search system, cover all chemically challenging cases, the assumptions made in UC2 cover most applicable cases in metabolomics. The existence of candidates from specified databases such as FlavonoidViewer for flavonoids and HMDB for human metabolites is useful information for metabolite annotation. An advantage of the system is that concise results can be obtained even when datasets are added in the future.

## Funding

This work was supported by the National Bioscience Database Center of Japan Science and Technology Agency [Project ID 14523923], the Kazusa DNA Research Institute and the Cooperative Research Project between the Kazusa DNA Research Institute, Japan and the National Institute of Agricultural Sciences of the Rural Development Administration of the Republic of Korea [Project No. PJ012099].

*Conflict of Interest*: none declared.

## Supplementary Material

Supplementary Data
