# Supplementary material for: UC2 search: using unique connectivity of uncharged compounds for metabolite annotation by database searching in mass spectrometry-based metabolomics
Source: Bioinformatics. 2017 Oct 12;34(4):698–700. doi: 10.1093/bioinformatics/btx649 (PMC5860614; doi:10.1093/bioinformatics/btx649)
Supplement: Supplementary Data [file btx649_supplementarydata.pdf]

Supplementary Material for

UC2 search: Using unique connectivity of uncharged compounds for metabolite annotation by database searching in mass spectrometry-based metabolomics

Nozomu Sakurai[1,*], Takafumi Narise[1], Joon-Soo Sim[2], Chang-Muk Lee[2], Chiaki Ikeda[1], Nayumi Akimoto[1] and Shigehiko Kanaya[3]

[1]Department of Technology Development, Kazusa DNA Research Institute, Kisarazu, Chiba 292-0818, Japan; [2]Department of Agricultural Biotechnology, National Institute of Agricultural Sciences, 370 Nongsengmyung-Ro, Jeonju 54874, Korea; [3]Graduate School of Information Science, Nara Institute of Science and Technology, 8916-5 Takayama, Ikoma, Nara 630-0192, Japan

*To whom correspondence should be addressed.

Contact: sakurai@kazusa.or.jp

1. Supplementary Results and Discussion

1.1. Practical examples showing issues in conventional searches and solving them using UC2

We describe here five examples that show the issues occurring in a search executed in the normal way (conventional search) and how to solve them by performing a search using the Unique Connectivity of Uncharged Compounds (UC2 search). For both searches, the MFSearcher graphical user interface (GUI) tool was used to obtain the search results and the same databases, namely, Kyoto Encyclopedia of Genes and Genomes (KEGG), KNApSAcK, a flavonoid database (hereafter referred to as FlavonoidViewer, http://metabolomics.jp/wiki/Category:FL), HMDB and LIPID MAPS were selected as target databases. Metabolites were assumed to be detected using liquid chromatography (LC)–mass spectrometry (MS) with electrospray ionization (ESI), which is often used in untargeted metabolome analyses.

1.1.1. False positives caused by entries registered as charged molecules in the databases

Most of the compounds in compound databases are registered as neutral molecules, whereas in LC–MS analyses, mass values (*m/z* value) of metabolites are measured as charged form, namely ions.

Therefore, to search for compounds that match the *m/z* value, it is essential to estimate the adduct of the detected ion. $[M]^+$, $[M+H]^+$, $[M+NH_4]^+$ and $[M+Na]^+$ are often observed in the positive mode where cations are detected, and $[M]^-$, $[M-H]^-$ and $[M+HCOO]^-$ are often observed in the negative mode where anions are detected. For example, when a peak is detected at an *m/z* value of 580.1575 in the positive mode, neutral compounds having mass values of 579.1502, 562.1237 or 557.1683 are searched for, which correspond to the estimated adduct ions $[M+H]^+$, $[M+NH_4]^+$ or $[M+Na]^+$, respectively. The detected mass value of 580.1575 is used to search the compounds that are registered as molecular ions ($[M]^+$). Therefore, estimation of the type of adduct ion has a large effect on the search results.

The type of adduct of the detected peak can be estimated based on mass differences between the peaks that were eluted simultaneously, because the variations of the adducts occur during ionization, which takes place just after the LC separation and just before the MS detection (**Supplementary Fig. S1**). For example, if the mass difference between a pair of co-detected peaks is exactly 21.9820, the theoretical mass difference between sodium and hydrogen, this strongly suggests that the peak with the larger *m/z* value should be $[M+Na]^+$ and the one of smaller *m/z* should be $[M+H]^+$. However, in practical LC–MS data, many peaks cannot be detected with co-detected peaks; therefore the adducts are not determined. In this case, we have to prioritise and choose some adduct ions from various possibilities considering the sample analysed and the chromatography conditions. Typically, a protonated ion $[M+H]^+$ or a deprotonated ion $[M-H]^-$ as ions ideally ionised without salts in the eluents are prioritised in the positive or negative mode, respectively.

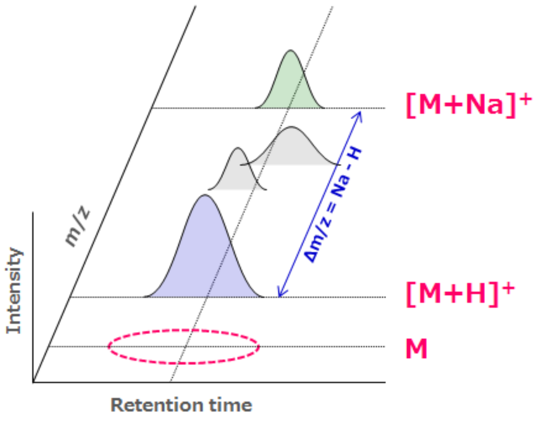

Supplementary Fig. S1. Determination of adduct ions.

When no co-detected peaks can be identified, we cannot judge whether the peak is detected as a molecular ion such as $[M]^+$ or a typical adduct ion such as $[M+H]^+$. Some metabolites can be charged themselves and detected as molecular ions, $[M]^+$ or $[M]^-$. Typical examples of molecular

ions are anthocyanins as pigment compounds in plants and quaternary ammonium compounds such as phosphatidylcholines. These compounds are usually registered as charged molecules in compound databases. As we show in **Supplementary Table S1**, a remarkable number (1.2–7.2%) of compounds in the databases are registered as charged molecules. Therefore, when no co-detected peaks can be identified, the abovementioned *m/z* value of 580.1575 should be searched as both a molecular ion ($[M]^+$) and a typical adduct ion ($[M+H]^+$). Searching for only one of them will be the cause of false negatives.

Here we show examples of issues caused by these two cycles of searching. An *m/z* value of 580.1575, detected in the positive mode by Iijima *et al.* (2008), was searched using the MFSearcher GUI tool assuming both $[M]^+$ and $[M+H]^+$. KEGG, KNApSAcK, HMDB, FlavonoidViewer and LIPID MAPS were used as the target databases. A 5 ppm mass tolerance was allowed. Twenty-nine candidates were found when a search was made for 580.1575 assuming $[M]^+$. When the neutralized mass value of 579.1503 for $[M+H]^+$ was searched, four candidates were found. Next, we checked the appropriateness of these candidates by considering their charges. The 29 candidates found when a search was made assuming $[M]^+$ have to be registered as $[M]^+$ in the databases too. We checked the structure of these candidates at the websites of the original compound databases and found that all 29 candidates were registered as neutral molecules. Therefore, these candidates were all false positives. Similarly, the four candidates found assuming $[M+H]^+$ were checked and they were also found to be false positives registered as $[M]^+$ (**Supplementary Fig. S2**).

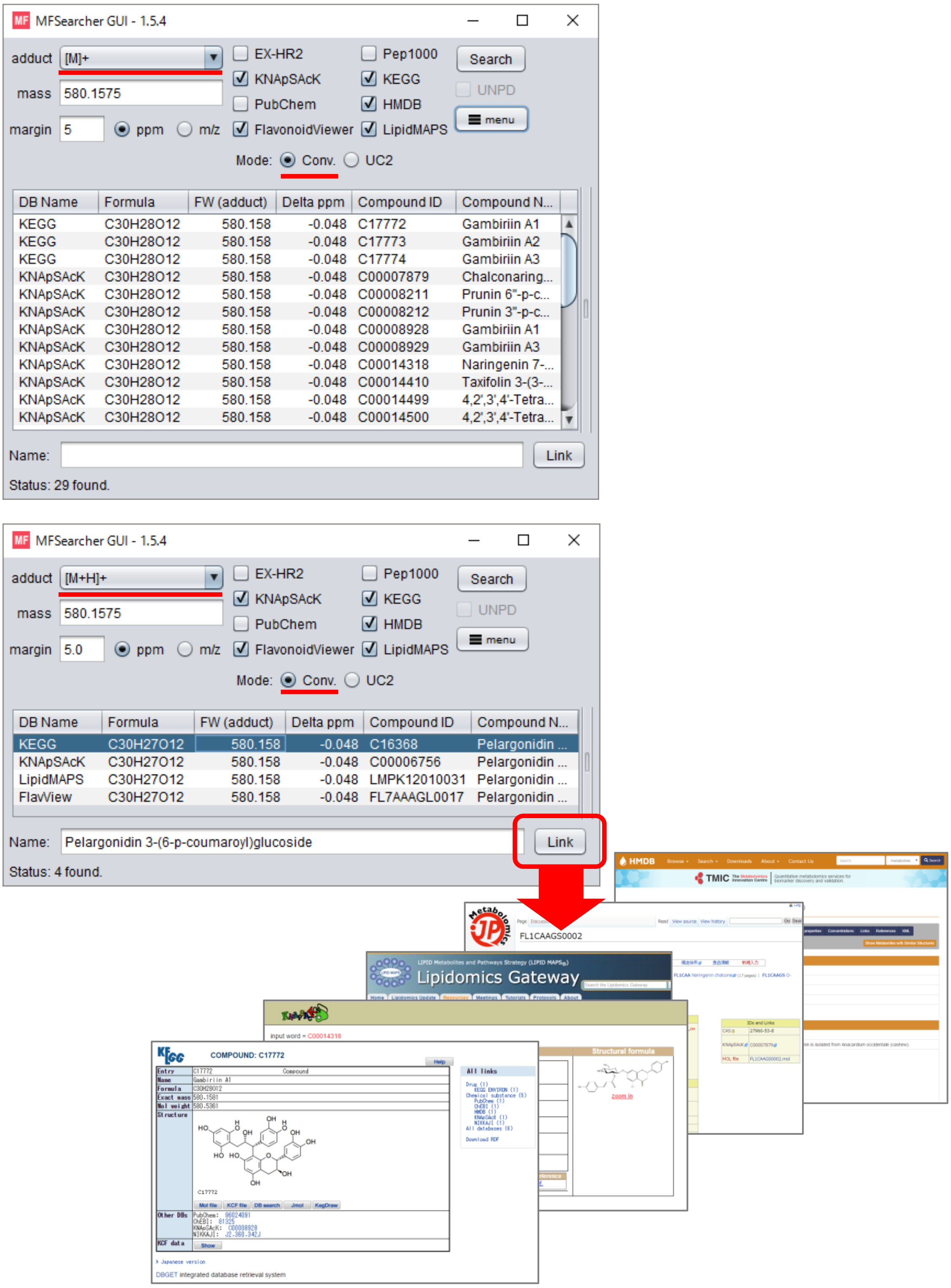

Supplementary Fig. S2. Two cycles of database searching and checking the records in a conventional search.

Using UC2, the false positives in the conventional search are eliminated. With UC2, charged compounds in the original databases are tentatively neutralized by adding or removing hydrogens to or from the formulae and the tentatively neutralized mass values are registered. Hydrogen is selected for adjustment of the neutralized mass, because in most cases, $[M+H]^+$ or $[M-H]^-$ are typical default adduct ions when no clues are obtained to estimate the true adduct ions.

When the detected mass, 580.1575, is searched as $[M+H]^+$ using UC2, compounds that have a neutralized mass value of 579.1502 are searched. Indeed, the correct result — in this case, no candidate — was obtained in the UC2 search with the same target databases (KEGG, KNApSAcK, LIPID MAPS, HMDB and FlavonoidViewer) as used in the conventional search (**Supplementary Fig. S3**).

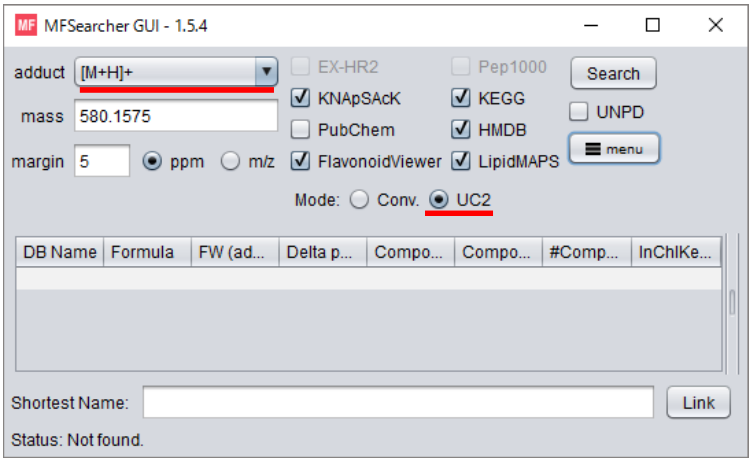

Supplementary Fig. S3. An example of UC2 search results. The false positives observed in the conventional search (**Supplementary Fig. S2**) were excluded.

In the next example, appropriate candidates are found. When a mass value of 859.21374 was applied in a conventional search, 12 candidates for $[M]^+$ and one candidate for $[M+H]^+$ were found. All these were found to have appropriate charges upon checking on the database websites. When the mass value was applied in a single UC2 search with $[M+H]^+$, the same 13 candidate compounds were found in five results consisting of the constitutional isomers (**Supplementary Fig. S4**, see **Supplementary Results and Discussion 1.1.3** for the details of the compiled results).

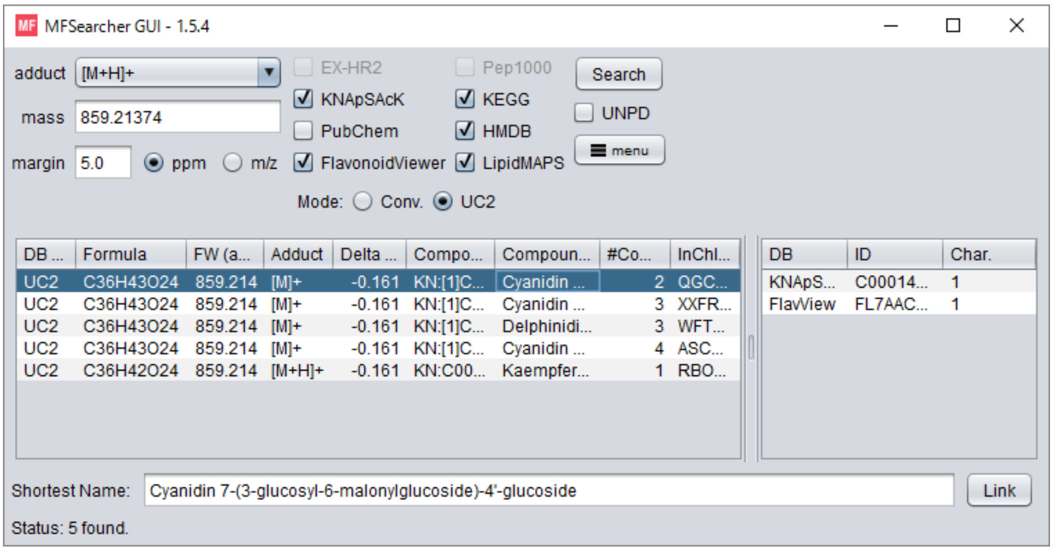

Supplementary Fig. S4. An example of UC2 search results. Candidates with appropriate charges are correctly obtained.

The elimination of apparent false positives with mismatching charge is a practical advantage of UC2, because researchers have to check the appropriateness of the candidates in a conventional search by checking the original databases one by one, as shown in these examples. Small numbers of candidates (30 and 13) are found here, but in practical data, more than 100 candidates will often be hit per query, as shown in **Supplementary Figs S12–14**. Many isomers are known in compound groups such as lipids and flavonoids (**Supplementary Table S1**, note the low proportion of unique formulae) and this would increase the number of candidates. Therefore, checking all candidates found in a conventional search with thousands of peaks detected in each LC–MS run is not practical. Using UC2 solves this issue. Among the databases used here, a search function that takes into account a match of charge is only provided by the LIPID MAPS website. KEGG and FlavonoidViewer do not even provide a web user interface to search compounds by mass values. Therefore, the unique cross-database search function using UC2 implemented in the MFSearcher web service and MFSearcher GUI tool should contribute to a better annotation of metabolites detected in untargeted metabolome analyses using LC–MS.

1.1.2. False positives caused by compounds registered as multiple components

When the *m/z* value of 939.2384, detected in the negative mode (Iijima *et al.*, 2008), was searched as $[M]^-$ in the conventional search, the compound Novaeguinoside B ($C_{36}H_{56}NO_{17}S_3Na_3$) was found in LIPID MAPS as a candidate. However, this is a false positive because the structure

excluding the sodium ions should be much smaller; e.g. 916.2511 as $[C_{36}H_{56}NO_{17}S_3+Na_2]^-$, 872.2872 as $[C_{36}H_{56}NO_{17}S_3+H_2]^-$ and 290.0908 as $[C_{36}H_{56}NO_{17}S_3]^{3-}$. Researchers have to check if the candidate is a salt or not by referring to the structural information such as provided on the original web page of the database. When the mass value was applied in a UC2 search with $[M\text{-}H]^-$, a correct result (no candidate) was obtained.

One advantage of the UC2 search is that both salt and non-salt entries are obtained in a compiled result. When another *m/z* value, 346.0558 detected in negative mode (Iijima *et al.*, 2008), was applied in a UC2 search with $[M\text{-}H]^-$, six results with the formula $C_{10}H_{14}N_5O_7P_1$ were found (**Supplementary Fig. S5**). One of the results, 'Adenosine monophosphate', included two KEGG entries (C00020 and C18344); the latter is a di-sodium salt of the former.

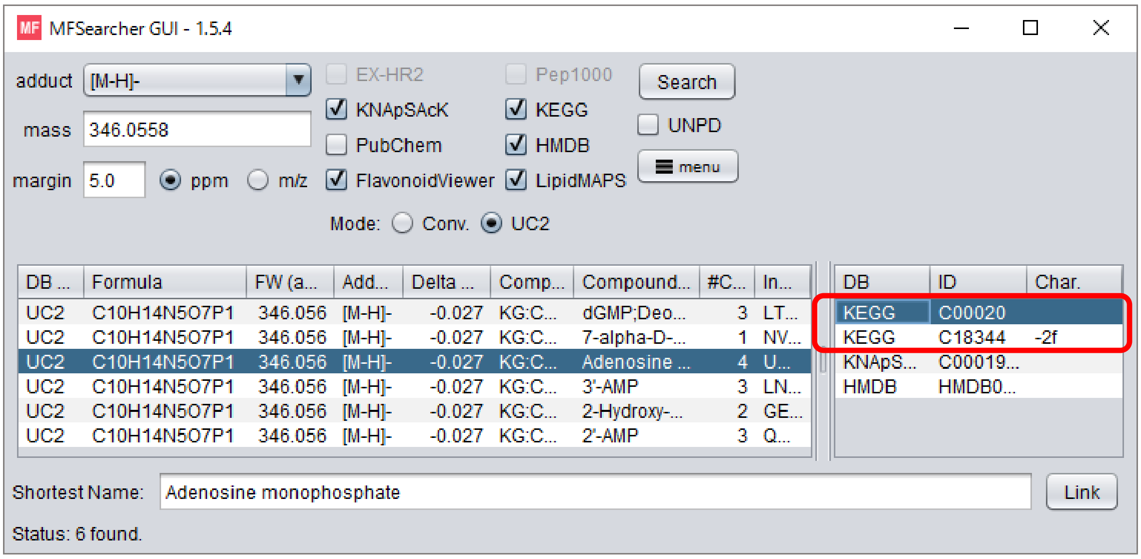

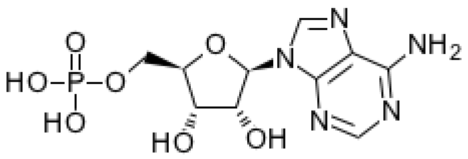

C00020

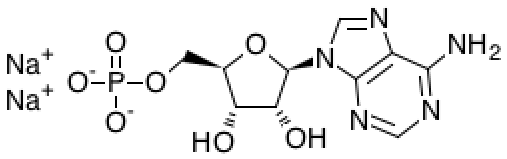

C18344

Supplementary Fig. S5. An example of UC2 search results. A component in the salt is correctly found.

It is an advantage of the UC2 search that the compounds registered only as salts among databases can be searched (**Supplementary Fig. S6**).

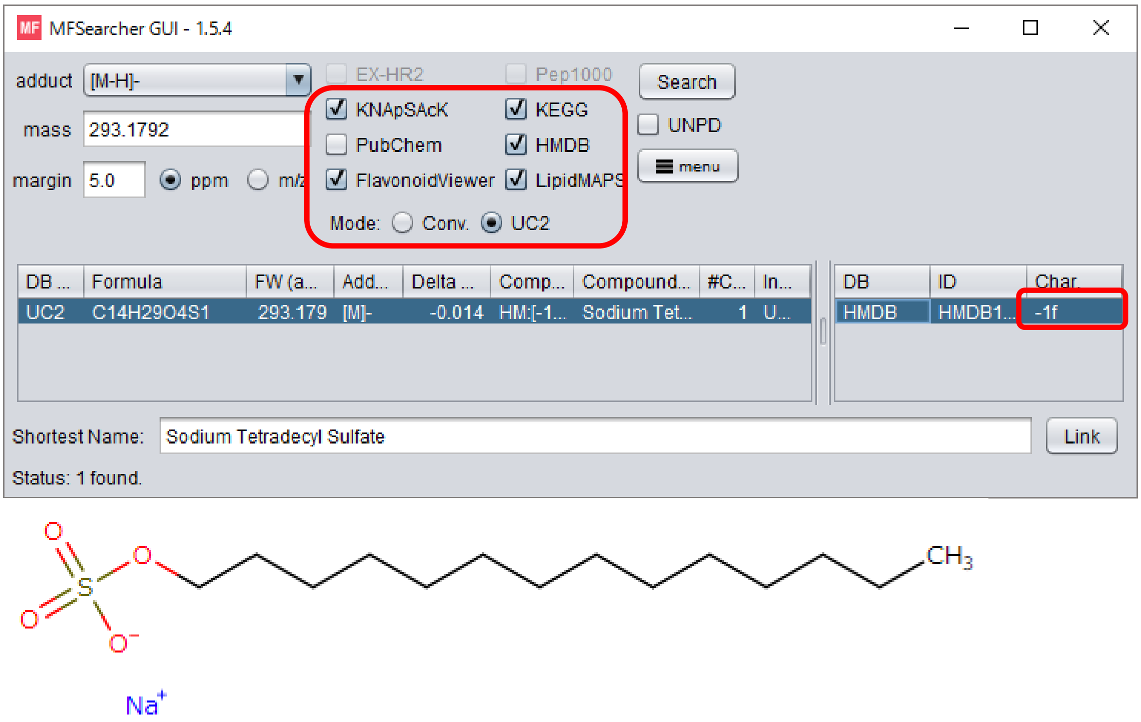

Supplementary Fig. S6. An example of UC2 search results. A compound registered only as salt is found.

1.1.3. Complexity of the search results caused by the existence of isomers

Here we exemplify a further advantage of UC2, namely that records with the same atomic connectivity are compiled in a single result. In a conventional search, 12 candidates including glutamine were obtained when the *m/z* value of 147.0764, detected in the positive mode (Iijima *et al.*, 2008), was searched as $[M+H]^+$ (**Supplementary Fig. S7**, upper panel). Judging from the compound names, the results seemed to contain stereoisomers of glutamine and other isomers such as 3-ureidoisobutyrate. Users have to rearrange the results to obtain the information they want. A typical interest of untargeted metabolome researchers is firstly the constitutional isomers and then the stereoisomers.

When the mass value was applied in a UC2 search, for the same formula ($C_5H_{10}N_2O_3$), four compiled results — representing four constitutional isomers — were obtained (**Supplementary Fig. S7**, lower panel). The shortest name among the compiled entries are displayed as a representative at the ‘Shortest Name’ field. In the result for ‘L-Glutamine’, three KEGG entries were found: L-Glutamine, D-Glutamine and a glutamine without stereochemistry information. L- and D-Glutamine were registered in HMDB and only L-Glutamine was registered in

KNApSAcK.

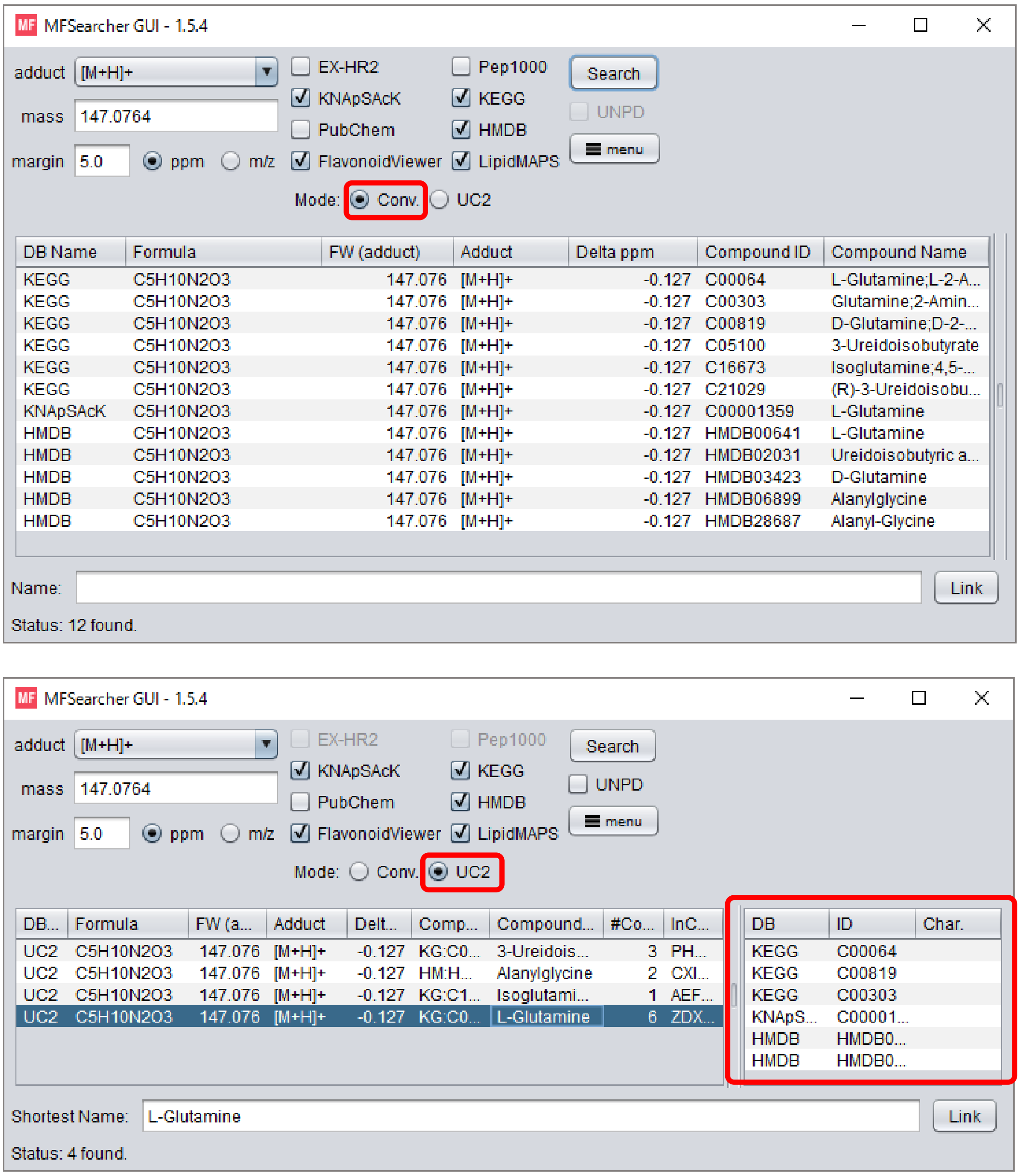

Supplementary Fig. S7. Difference between the results of a conventional search (upper panel) and a UC2 search (lower panel). In the UC2 search results, the same constitutional isomers are compiled in a single result, and the stereoisomers are included in each result.

As shown in this example, compiling the constitutional isomers in the results of the UC2 search should be helpful for understanding the search results. In the example, all four constitutional isomers have chiral carbons. However, stereoisomers are registered in the compound databases only for glutamine and 3-ureidoisobutyrate. UC2 masks this incompleteness of database registration and provides constitutional isomers first.

1.1.4. Obtaining candidates for different dissociation forms by the UC2 search

When a mass value of 496.3398 was applied in a conventional search, two formulae ($C_{24}H_{51}NO_7P$, one entry each in KEGG and HMDB, and $C_{28}H_{48}O_7$, three entries in KEGG) were obtained as $[M]^+$ and a single formula ($C_{24}H_{50}NO_7P$, five entries in LipidMAPS, one entry in HMDB) was found as $[M+H]^+$. When searched as $[M+H]^+$ using UC2, four constitutional isomers ($C_{24}H_{50}NO_7P$) were obtained, and the abovementioned candidate $C_{28}H_{48}O_7$ was found as a false positive with mismatching charge. Among the four true positive candidates with $C_{24}H_{50}NO_7P$, one result including the KEGG entry C04102 was a kind of glycerophosphocholine, a quaternary ammonium compound. In KEGG, this compound is registered as its $[M]^+$ form, while the compound is registered as the neutral form in LIPID MAPS and HMDB (**Supplementary Fig. S8**). As shown in this example, UC2 can provide the candidates registered in different dissociation forms in a single result.

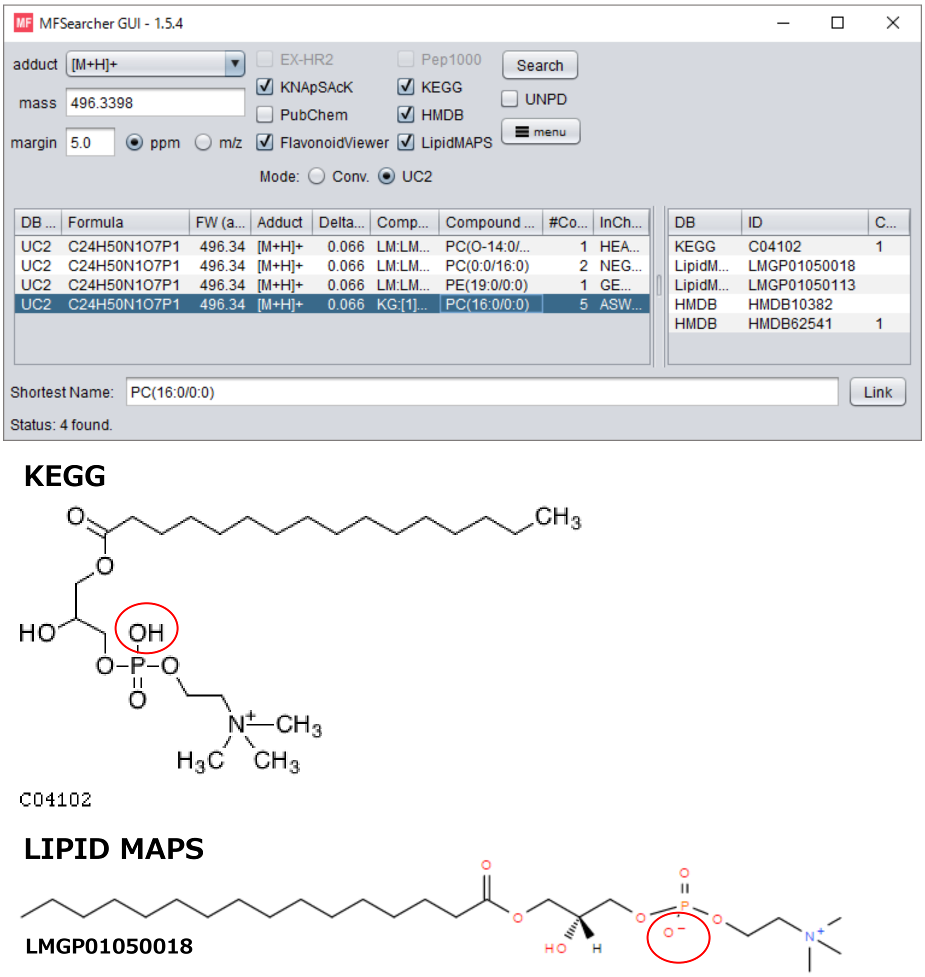

Supplementary Fig. S8. An example of UC2 search results. Candidates registered in different dissociation forms can be searched and compiled in one result.

1.1.5. Application of UC2 to hardly neutralizable charged compounds

When a mass value of 174.1489 was applied in a conventional search, six candidates including Muscarine and Butyrylcholine were found as $[M]^+$ and two candidates, 9-Amino-nonanoic acid and 3R-Aminononanoic acid, were obtained as $[M+H]^+$. Judging by their charge, these eight candidates were true positives. When the mass value was searched by UC2 with $[M+H]^+$, four results including all these eight candidates were also obtained. Butyrylcholine seems hard to neutralize and a dehydrogenated form of this compound would not be expected. Nevertheless, the compound is correctly searched by UC2 where the positive and negative charges were forcedly neutralized by removing and adding equivalent numbers of hydrogens from and to the formula to adjust the mass differences between $[M]^+$ and $[M+H]^+$ (**Supplementary Fig. S9**). This result shows that UC2 is applicable to searches for hardly neutralizable compounds; the adjustment of the charge by tentatively adding or removing hydrogen atoms to or from the formula is a practical procedure.

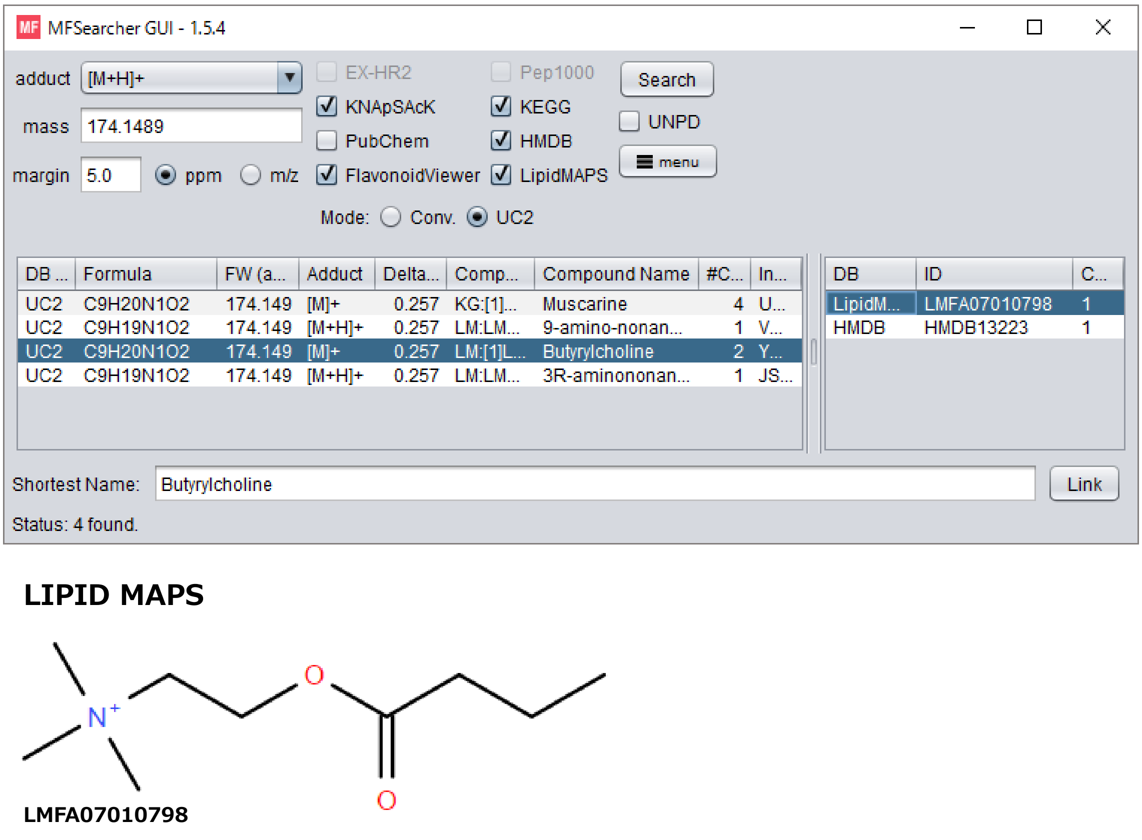

Supplementary Fig. S9. An example of UC2 search results. Compounds such as Butyrylcholine that are not expected to be neutralized in normal conditions can be searched correctly.

1.2. Example of the records of the UC2 database

In the UC2 database, the IDs of the compounds in each compound database are stored based on InChIKey skeletons (the first block in the hash of the IUPAC International Chemical Identifier). **Supplementary Fig. S10** is an example of the HMDB records stored in the MariaDB RDB system (MariaDB Foundation) in the MFSearcher web service. A tentatively neutralized mass value, a tentatively neutralized formula, IDs and the name of the compound are associated with an InChIKey skeleton. Multiple IDs can be assigned to the same InChIKey skeleton. The shortest name among the associated compounds is used as the representative. Signatures at the head of compound IDs represent the compound databases: KG, KEGG; KN, KNApSAcK; FL, FlavonoidViewer; HM, HMDB; LM, LIPID MAPS; UN: UNPD; and PC, PubChem. The number, 'f' and 'r' in the square brackets represent the charge, whether the compound is registered as multiple components and whether the compound is registered as radical. Complete data for HMDB and PubChem are available on the MFSearcher website (http://webs2.kazusa.or.jp/mfsearcher/uc2/).

Tentatively Neutralized Mass	InChIKey skeleton	Tentatively Neutralized Formula	IDs	Shortest Name
2.01565006	UFHFLCQGNIYNRP	H2	HM:HMDB01362	Hydrogen
4.00260000	SWQJXJOGLNCZEY	He	HM:HMDB37238	Helium
16.03130013	VNWKTOKETHGBQD	CH4	HM:HMDB02714	Methane
17.02654910	QGZKDVFQNNGYKY	H3N	HM:HMDB00051,HM:[1]HMDB41827	Ammonia
18.01056469	XLYOFNOQVPJJNP	H2O	HM:[-1]HMDB01039,HM:HMDB02111	Water
20.00622823	KRHYYFGTRYWZRS	HF	HM:[-1]HMDB00662	Fluoride
26.98153900	XAGFODPZIPBFFR	Al	HM:HMDB15456	Aluminium
27.01089904	LELOWRISYMNNSU	CHN	HM:HMDB60292	Hydrogen cyanide
27.99491462	UGFAIRIUMAVXCW	CO	HM:[r]HMDB01361	Carbon monoxide
28.00614801	IJGRMHOSHXDMSA	N2	HM:HMDB01371	Nitrogen
28.03130013	VGGSQFUCUMXWEO	C2H4	HM:HMDB29594	Ethylene
29.99798863	MWUXSHHQAYIFBG	ON	HM:[r]HMDB03378	Nitric oxide
30.01056469	WSFSSNUMVMOOMR	CH2O	HM:HMDB01426	Formaldehyde
31.04219917	BAVYZALUXZFZLV	CH5N	HM:HMDB00164,HM:HMDB60291	Methylamine
31.98982924	MYMOFIZGZYHOMD	O2	HM:HMDB01377	Oxygen
32.02621475	OKKJLVBELUTLKV	CH4O	HM:HMDB01875	Methanol
32.03744814	OAKJQQAXSVQMHS	H4N2	HM:HMDB12973	Hydrazine
32.99765428	OUUQCZGPVNCOIJ	HO2	HM:[-1 r]HMDB02168	Superoxide
33.02146373	AVXURJPOCDRRFD	H3ON	HM:HMDB03338	Hydroxylamine
33.02146373	GSWAOPJLTADLTN	H3ON	HM:HMDB32439	Nitrogen oxides
33.98772106	UCKMPCXJQFINFW	H2S	HM:[-2]HMDB00598	Sulfide
33.98772106	RWSOTUBLDIXVET	H2S	HM:HMDB03276	Hydrogen sulfide
33.99723810	XYFCBTPGUUZFHI	H3P	HM:HMDB34790	Phosphine
34.00547931	MHAJPDPJQMAIIY	H2O2	HM:HMDB03125	Hydrogen peroxide
35.97667813	VEXZGXHMUGYJMC	HCl	HM:[-1]HMDB00492,HM:HMDB02306	Chloride ion
39.96238400	XKRFYHLGVUSROY	Ar	HM:HMDB37240	Argon
39.97995662	CPLXHLVBOLITMK	OMg	HM:HMDB15458	Magnesium oxide
41.02654910	WEVYAHXRMPXWCK	C2H3N	HM:HMDB61869	Acetonitrile
43.00581366	XLJMAIOERFSOGZ	CHON	HM:[-1]HMDB02078	Cyanate
43.98982924	CURLTUGMZLYLDI	CO2	HM:HMDB01967	Carbon dioxide
44.00106264	GQPLMRYTRLFLPF	ON2	HM:HMDB35807	Nitrous oxide
44.00695928	VGTPKLINSHNZRD	HO2B	HM:HMDB34769	Boric acid (HBO2)
44.02621475	IKHGUXGNUITLKF	C2H4O	HM:HMDB00990	Acetaldehyde
44.06260026	ATUOYWHBWRKTHZ	C3H8	HM:HMDB31630	Propane
45.02146373	ZHNUHDYFZUAESO	CH3ON	HM:HMDB01536	Formamide
45.05784923	QUSNBJAOOMFDIB	C2H7N	HM:HMDB13231	Ethylamine
45.05784923	ROSDSFDQCJNGOL	C2H7N	HM:HMDB00087	Dimethylamine
46.00547931	BDAGIHXWWSANSR	CH2O2	HM:HMDB00142	Formic acid

Supplementary Fig. S10. An example of records in the UC2 database in the MFSearcher web service. The head of the data in the table for HMDB is shown.

1.3. Features of the entries in the databases

The number of charged entries and unique connectivities differs very much between the compound databases; therefore, a query of multiple databases with the proper charge is needed to obtain the maximum number of appropriate candidate compounds. **Supplementary Table S1** shows a summary of the entries in the compound databases. A substantial number of compounds were stored as charged molecule in each database, especially in FlavonoidViewer (7.2%). As for the charged compounds in FlavonoidViewer, LIPID MAPS and KNApSAcK, less than 2% were those whose uncharged counterparts with the same connectivity were also registered in the same database (**Supplementary Table S2**). This suggests that most charged compounds are registered only as their

charged form and are not registered redundantly as neutral form; therefore, multiple searches assuming the molecular ions such as $[M]^+$ and $[M]^-$ and adduct ions such as $[M+H]^+$ and $[M-H]^-$ are essential to obtain appropriate candidates from these databases (see **Supplementary Results and Discussion 1.1.4**). Relatively low ratios of the unique InChIKey skeletons were found in databases with a huge number of entries (UNPD and PubChem, 73% and 78%, respectively), implying that many entries with different constitutional isomers and stereoisomers are registered in them (**Supplementary Table S1**). Low ratios of unique formulae in FlavonoidViewer (15.2%), LIPID MAPS (18.3%), UNPD (14.2%) and PubChem (2.7%) suggest that many isomeric and/or fragmented compounds are included there (**Supplementary Table S1**). **Supplementary Table S3** shows the extent of the shared unique connectivity (InChIKey skeletons) between the databases. Large portions of unique InChIKey skeletons are shared between the largest databases (UNPD and PubChem). Most of the unique InChIKey skeletons in FlavonoidViewer are shared with LIPID MAPS and KNApSAcK (95% and 100%, respectively), because the entries in FlavonoidViewer have been incorporated into LIPID MAPS and KNApSAcK. On the other hand, in the other databases except these three (KEGG, LIPID MAPS, HMDB and KNApSAcK), the proportion of shared unique InChIKey skeletons is less than 34%, suggesting that unique compounds are stored in these databases. Especially in HMDB, 37% are shared and hence 63% are unique even when compared with UNPD and PubChem. These results suggest that it is necessary for researchers to query multiple databases to cover the maximum number of compounds and also to remove the redundancy of the same compounds between databases.

2. Supplementary Methods

2.1. Details of the data acquired from the compound databases

The structural data of compounds were obtained from the compound database of the Kyoto Encyclopedia of Genes and Genomes (KEGG) (Kanehisa *et al.*, 2016), KNApSAcK (Afendi *et al.*, 2012), a flavonoid database (hereafter referred to as FlavonoidViewer, http://metabolomics.jp/wiki/Category:FL), LIPID MAPS (Fahy *et al.*, 2009), Human Metabolome Database (HMDB) (Wishart *et al.*, 2013), UNPD (Gu *et al.*, 2013) and PubChem (Wang *et al.*, 2009) as MDL Mol files or SDF files. The download dates or the dataset versions are as follows: KEGG, downloaded on June 28th, 2017; KNApSAcK, provided by Dr. Shigehiko Kanaya on March 17th, 2015; FlavonoidViewer, retrieved on August 20th, 2015; LIPID MAPS, the file LMSDFDownload6Dec16.zip was downloaded; HMDB, version 3.6 released on June 11th, 2017; UNPD, downloaded on August 20th, 2015 and PubChem, downloaded on June 27th 2017.

2.2. Construction of the UC2 database

The Chemistry Development Kit (CDK, version 2.0) (Willighagen *et al.*, 2017) and the Java Development Kit (JDK, version 1.7, Oracle Corporation) were used for molecular calculations and generation of the IUPAC International Chemical Identifier (InChI) and the hash of the InChI (InChIKey) (Heller *et al.*, 2013) as follows: Entries for which the registered formula did not match the formula calculated from the structure data were excluded because they were considered as misregistrations in the original databases. When multiple components were included in a record (hereafter referred to as fragmented records), the one with the largest molecular weight was used as a representative. After the implicit hydrogens were added, a Kekulé representation was assigned to the aromatic systems and the standard InChIKey and molecular formula were calculated. Entries to which implicit hydrogens could not be added were excluded. Compounds in PubChem with molecular weight larger than 5000 were excluded to reduce the calculation time. Isotopic compounds were excluded. The charge of the molecules was detected by summing the charges of the atoms. The tentatively neutralized formulae of the molecules were obtained by adding or removing hydrogens to or from the formula. Namely, an equivalent number of hydrogens was removed from the formulae for positively charged molecules, and an equivalent number of hydrogens was added to the formulae for the negatively charged ones. For example, in the case of tetramethylamine ($C_4H_{12}N^+$), a hydrogen was subtracted to give $C_4H_{11}N$ as neutralized formula. InChIKey skeletons should not be changed by this manipulation of hydrogens in most cases; therefore, they can be used as unique signatures for constitutional isomers regardless of their charged states (**Supplementary Results and Discussion 1.1.2 and 1.1.4**), although they might be changed in some cases such as some specific tautomers. Records with an insufficient number of hydrogens for this formula manipulation were excluded. The method of CDK and Java source codes used in the molecular calculations is shown in **Supplementary Fig. S11**. The first block (14 letters) of InChIKey (hereafter referred to as the InChIKey skeleton) was used as a unique signature for the same connectivity of atoms (Heller *et al.*, 2013). The mass values were calculated based on the exact mass values of the atoms published by IUPAC (de Laeter *et al.*, 2003). To recognise the source of the original database and if the records in the original database were charged, contained multiple components and were radicals, we attached labels comprising the signature of the database, charge, ‘f’ and ‘r’ for each compound ID. Signatures of the compound databases are as follows: KG, KEGG; KN, KNApSAcK; FL, FlavonoidViewer; HM, HMDB; LM, LIPID MAPS; UN, UNPD and PC, PubChem. Examples of the labelled IDs in HMDB are shown in **Supplementary Fig. S10**. The InChIKey skeleton, the tentatively neutralized formula and its mass value, the original IDs with labels and the shortest name among the associated compounds as representative were stored in

MariaDB (5.0.77, MariaDB Foundation) on a Red Hat Enterprise Linux Server 7.1 (the UC2 database). The web service to search the UC2 database was constructed with Apache Tomcat and implemented in MFSearcher (http://webs2.kazusa.or.jp/mfsearcher) (Sakurai *et al.*, 2013) running on the server. The GUI tool for searching the UC2 database was developed with JDK 1.7.

```
// The MDMolecule object was generated from MDL Mol file or SDF file
// obtained from the compound databases

MDMolecule mol;

// Addition of implicit hydrogens

AtomContainerManipulator.percieveAtomTypesAndConfigureAtoms( mol );
CDKHydrogenAdder adder = CDKHydrogenAdder.getInstance( mol.getBuilder() );
adder.addImplicitHydrogens(mol);
AtomContainerManipulator.convertImplicitToExplicitHydrogens(mol);

// Obtaining components

if( ! ConnectivityChecker.isConnected( mol ) ){
   IAtomContainerSet components = ConnectivityChecker.partitionIntoMolecules( mol );
}

// Detection of isotopes
boolean containsIsotope = false;
IsotopeFactory ifc = Isotopes.getInstance();
for( IAtom atom: mol.atoms() ){
   ifc.configure( atom );
   double massCurrentAom = atom.getExactMass();
   double massMajorIsotope = ifc.getMajorIsotope( atom.getSymbol() ).getExactMass();
   if( massCurrentAtom != massMajorIsotope ){
      containsIsotope = true;
      break;
   }
}
```

```
// Kekulization

Kekulization.kekulize( mol );

// Generation of InChI, InChIKey and InChIKey skeleton

InChIGeneratorFactory f = InChIGeneratorFactory.getInstance();
InChIGenerator gen = f.getInChIGenerator( mol );

String inchi = gen.getInchi();
String inchikey = gen.getInchiKey();
String inchikeySkeleton = inchikey.substring(0,14);

// Calculation of the charge of the molecule

int charge = 0;
for(IAtom atom: mol.atoms()){
   if(atom.getFormalCharge() != 0){
      charge += atom.getFormalCharge();
   }
}

// Detection of the radicals

boolean isRadical = false;
for( ISingleElectron se: mol.singleElectrons() ){
   isRadical = true;
   break;
}
```

Supplementary Fig. S11. The methods of CDK used for molecular calculations to construct the UC2 database.

2.3. Preparation of the metabolite peak lists

2.3.1. Metabolites list in tomato fruits

As an example of manually curated metabolite peaks including secondary metabolites biosynthesized in plants, we used a list of 869 metabolite peaks detected and annotated in tomato fruits using LC-Fourier transform ion cyclotron (FT-ICR) MS (Iijima *et al.*, 2008). The list — provided as Supplementary Table S2 of Iijima *et al.* (2008) — included 510 metabolites that were detected in the positive mode of electron spray ionization (ESI) and 519 metabolites detected in the ESI negative mode. A set of 160 metabolites was detected in both modes, and 350 and 359 metabolites were detected only in the positive or the negative mode, respectively.

2.3.2. Metabolites list in human urine

As an example of a computationally calculated and not curated peak list, we chose data obtained from human urine. Lists of metabolite peaks in human urine were prepared from the raw data published by van der Hooft *et al.* (2016) as follows: The raw data analysed in ESI positive mode (Pooled_Urine_15_POS.raw) and data analysed in ESI negative mode (Pooled_Urine_14_NEG.raw) using LC-Orbitrap MS in the study MTBLS307 in MetaboLights (Salek *et al.*, 2013) were downloaded. The raw data were converted to mzXML files using ProteoWizard software (version 3.0.6447) (Kessner *et al.*, 2008). Detection of the metabolite peaks and estimation of the adducts were performed using an in-house version of the PowerGet software (Sakurai *et al.*, 2014) that was slightly modified for batch processing. Sets of 1,264 and 1,475 metabolite peaks were detected in the positive and negative modes, respectively.

2.3.3. Random mass list

To examine the effect of biological selection of the metabolites on the database search results, a random mass list in the *m/z* range of 100–1,500 was computationally generated using JDK 1.7. In total 6,491 and 6,379 mass values were generated to obtain exactly 1,000 mass values that showed results in database searches of the positive and negative modes, respectively.

2.4. Comparison of search results from the UC2 database and other compound databases

We compared the results from a search using UC2 (UC2 search) and a search in the normal way (conventional search). For both searches, the MFSearcher web service (http://webs2.kazusa.or.jp/mfsearcher) (Sakurai *et al.*, 2013) was used to get the search results and KEGG, KNApSAcK, FlavonoidViewer, LIPID MAPS and HMDB were selected as target databases. A protonated cation ($[M+H]^+$) and a deprotonated anion ($[M-H]^-$)

were assumed for the searches with randomly generated masses in the positive and negative modes, respectively. The neutralized mass values based on the estimated adduct ions were used for both the conventional and the UC2 search. In the conventional search, searches with the detected *m/z* values were also performed for the peaks of the default adduct ion ($[M+H]^+$ and $[M-H]^-$ for the positive and negative modes, respectively) and the results were merged to those obtained with the neutralized mass values. A mass tolerance of 5 ppm was allowed. In-house programs supporting an automatic search for a large number of mass values with the MFSearcher and for counting and compiling the results were written in Java and Perl. The causes of unusual results in queries, namely those queries whose results were only found in either the UC2 search or the conventional search, were manually investigated.

3. Supplementary Tables

Supplementary Table S1. Summary of the entries in the compound databases. Values mentioned in the Supplementary Results and Discussion are highlighted as bold face in red.

	Flavonoid Viewer [a]	KEGG	LIPID MAPS	HMDB	KNApSAcK	UNPD	PubChem
# Entries	6,864	15,864	40,159	74,263	50,692	229,004	91,385,223
# Fragmented Entries	0	432	10	139	0	0	4,416,521
# Charged Entries	492	601	475	871	750	3,547	2,728,406
# Unique Formula Neutralized	1,043	8,470	7,344	10,067	13,522	32,629	2,505,657
# Unique InChIKey	6,817	15,622	40,124	74,009	49,660	228,617	87,022,085
# Unique InChIKey Skeleton	6,415	14,363	36,549	68,526	45,508	167,096	71,565,070
	Ratio to # Entries						
Fragmented Entries	0.0%	2.7%	0.0%	0.2%	0.0%	0.0%	4.8%
Charged Entries	**7.2%**	3.8%	1.2%	1.2%	1.5%	1.5%	3.0%
Unique Formula Neutralized	**15.2%**	53.4%	**18.3%**	13.6%	26.7%	**14.2%**	**2.7%**
Unique InChIKey	99.3%	98.5%	99.9%	99.7%	98.0%	99.8%	95.2%
Unique InChIKey Skeleton	93.5%	90.5%	91.0%	92.3%	89.8%	**73.0%**	**78.3%**

[a] Flavonoid database at metabolomics.jp (http://metabolomics.jp/wiki/Category:FL)

Supplementary Table S2. Ratio of the charged entries whose uncharged counterpart with the same connectivity is registered in the same database. Values mentioned in the Supplementary Results and Discussion are shown as bold face in red.

	# Charged entry	# Uncharged entries that have the same connectivity as the charged entry	
FlavonoidViewer	492	0	**(0%)**
KEGG	601	86	(14%)
LIPID MAPS	475	8	**(2%)**
HMDB	871	156	(18%)
KNApSAcK	750	7	**(1%)**
UNPD	3,547	508	(14%)
PubChem	2,728,406	938,084	(34%)

Supplementary Table S3. Summary of the shared unique connectivities (InChIKey skeletons) in the databases. Parentheses show the ratio to the number of unique InChIKey Skeletons in the database. Values mentioned in the Supplementary Results and Discussion are shown as bold face in red.

	# in the database	# Found in other databases									
		Flavonoid Viewer [a]	KEGG	LIPID MAPS	HMDB	KNApSAcK	UNPD	PubChem	All [b]	All-P [b]	All-PU [b]
**Unique InChIKey Skeleton**											
**FlavonoidViewer [a]**	6,415	-	495 (8%)	**6,104 (95%)**	1,013 (16%)	**6,390 (100%)**	5,626 (88%)	6,325 (99%)	**6,414 (100%)**	6,413 (100%)	6,406 (100%)
**KEGG**	14,363	495 (3%)	-	**2,400 (17%)**	**4,661 (32%)**	**4,920 (34%)**	6,768 (47%)	14,082 (98%)	14,192 (99%)	9,364 (65%)	8,183 (57%)
**LIPID MAPS**	36,549	6,104 (17%)	**2,400 (7%)**	-	**8,155 (22%)**	**7,395 (20%)**	9,175 (25%)	36,522 (100%)	36,544 (100%)	15,958 (44%)	14,380 (39%)
**HMDB**	68,526	1,013 (1%)	**4,661 (7%)**	**8,155 (12%)**	-	**5,002 (7%)**	10,663 (16%)	24,665 (36%)	**25,362 (37%)**	18,131 (26%)	13,655 (20%)
**KNApSAcK**	45,508	6,390 (14%)	**4,920 (11%)**	**7,395 (16%)**	**5,002 (11%)**	-	39,660 (87%)	40,681 (89%)	43,585 (96%)	41,003 (90%)	13,407 (29%)
**UNPD**	167,096	5,626 (3%)	6,768 (4%)	9,175 (5%)	10,663 (6%)	39,660 (24%)	-	125,419 (75%)	128,800 (77%)	48,606 (29%)	
**PubChem**	71,565,070	6,325 (0%)	14,082 (0%)	36,522 (0%)	24,665 (0%)	40,681 (0%)	125,419 (0%)	-	169,346 (0%)		
									# Total unique InChIKey Skeleton		
									71,652,103	256,379	137,889

[a] Flavonoid database at metabolomics.jp (http://metabolomics.jp/wiki/Category:FL)

[b] Number of unique InChIKey skeletons found in one of the other databases (All), all other databases except PubChem (All-P), and all other databases except PubChem and UNPD (All-PU).

Supplementary Table S4. Summary of the database search results with conventional search and UC2 search. Values mentioned in the Results and Discussion are shown as bold face in red.

	Label	Tomato [a]		Urine		Random		Tomato [a]		Urine		Random		Calculation
		Positive	Negative	Positive	Negative	Positive	Negative	Positive	Negative	Positive	Negative	Positive	Negative	
		Number of queries						Ratio [b]						
# total queries	A	510	359	1264	1475	6491	6379	100%	100%	100%	100%	100%	100%	A/A
# queries whose results were found in conventional search or UC2 search	B	277	167	967	1092	1000	1000	54%	47%	77%	74%	15%	16%	B/A
# queries whose results were found in...														
conventional search	C	277	164	967	1091	998	984	54%	46%	77%	74%	15%	15%	C/A
1) with the neutralized mass values [c]	C1	226	138	903	1022	564	552	82%	84%	93%	94%	57%	56%	C1/C
2) with the detected or the randomly generated *m/z* values [d]	C2	134	57	182	230	563	539	48%	35%	19%	21%	56%	55%	C2/C
UC2 search	D	220	139	906	1012	556	553	43%	39%	72%	69%	9%	9%	D/A
conventional search and UC2 search	E	220	136	906	1011	554	537	43%	38%	72%	69%	9%	8%	E/A
# queries with a smaller number of results given in...														
conventional search	F	0	3	8	4	2	1	0%	2%	1%	0%	0%	0%	F/C
UC2 search	G	160	93	684	816	279	227	**73%**	**67%**	**75%**	**81%**	50%	41%	G/D
# queries with unique results that were found in...														
conventional search	H	63	38	119	118	332	376	23%	23%	12%	11%	33%	38%	H/C
UC2 search	I	74	42	163	177	276	292	**34%**	**30%**	**18%**	**17%**	50%	53%	I/D
								12%	11%	9%	8%	5%	6%	H/A
								15%	12%	13%	12%	4%	5%	I/A
# queries in UC2 search results that contained...														
modified entries (charged, fragmented, or radical)	J	49	25	146	224	31	27	22%	18%	16%	22%	6%	5%	J/D
charged	J1	46	23	127	206	27	25	21%	17%	14%	20%	5%	5%	J1/D
only charged entries were hit in the same database	J2	43	20	60	29	25	25	93%	87%	47%	14%	93%	100%	J2/J1
fragmented	J3	6	7	36	152	5	5	3%	5%	4%	15%	1%	1%	J3/D
radical	J4	0	0	0	2	0	0	0%	0%	0%	0%	0%	0%	J4/D
# queries whose results were found only in conventional search...	K	57	28	61	80	444	447	**20.6%**	**16.8%**	**6.3%**	**7.3%**	44.4%	44.7%	K/B
1) with the neutralized mass values [c]	L	7	2	3	13	20	16	12.3%	7.1%	4.9%	16.3%	4.5%	3.6%	L/K
false positives matched to the charged entries	L1	7	2	3	13	19	16	**100%**	**100%**	**100%**	**100%**	95%	100%	L1/L
false positives matched to the fragmented entries	L2	0	0	1	1	2	0	0%	0%	33%	8%	10%	0%	L2/L
2) with the detected or the randomly generated *m/z* values [d]	M	51	27	58	78	431	441	**89%**	**96%**	**95%**	**98%**	97%	99%	M/K
false positives matched to the uncharged entries	M1	51	21	58	77	427	428	**100%**	**78%**	**100%**	**99%**	99%	97%	M1/M
false positives matched to the fragmented entries	M2	0	1	1	1	1	3	0%	4%	2%	1%	0%	1%	M2/M
false positives matched to records of inappropriate charge [e]	M3	0	7	3	1	4	13	**0%**	**26%**	**5%**	**1%**	1%	3%	M3/M
# queries whose results were found only in UC2 search	N	0	3	0	1	2	16	**0.0%**	**1.8%**	**0.0%**	**0.1%**	0.2%	1.6%	N/B
true positives matched to the modified entries	N1	0	3	0	1	1	14	-	100%	-	100%	50%	88%	N1/N
false positives caused by miss registration in the database	N2	0	0	0	0	0	1	-	0%	-	0%	0%	6%	N2/N
false positives matched to records with repeat structure [f]	N3	0	0	0	0	1	1	-	0%	-	0%	50%	6%	N3/N
# queries where results excluding apparent false positives were found in...														
conventional search	O	220	136	906	1,011	554	537							C - K
UC2 search	P	220	139	906	1,012	555	551							D - (N2 + N3)

[a] Metabolites (160 peaks) detected in both positive and negative modes are shown as the positive.
[b] The ratios were calculated by division of the values given in the column 'Calculation' where values shown in the column 'Label' were used.
[c] $[M+H]^+$ and $[M-H]^-$ were used for the search with randomly generated mass values.
[d] The search was performed when the estimated adduct contained proton(s) as sole charged moiety.
[e] Inappropriate polarity or valence.
[f] Chemical structure with repeat units (e.g., C02072 in KEGG database) whose mol file is written as unrepeated structure.

4. Other Supplementary Figures

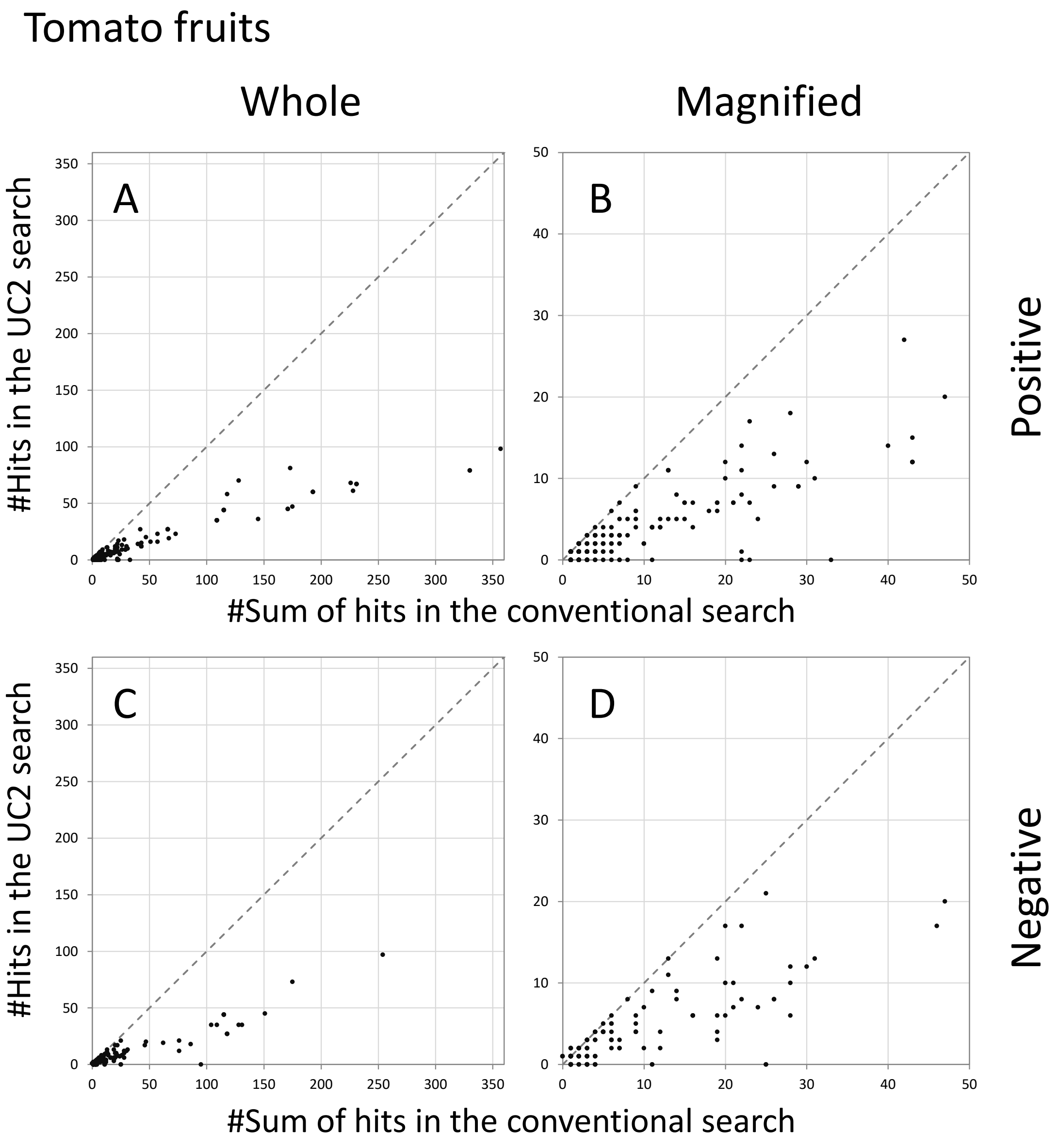

Supplementary Fig. S12. Comparison of the number of hits in the conventional search and the UC2 search for metabolites in tomato fruits. The number of compound hits in conventional search and in the UC2 search using the databases (KEGG, KNApSAcK, FlavonoidViewer, HMDB and LIPID MAPS) is plotted. The sum of search results with neutralized mass value and detected *m/z* value was used in the results of the conventional search. The mass values of the metabolite peaks detected in tomato fruits in positive (A and B) and negative (C and D) modes were searched with 5 ppm mass tolerance. B and D are magnified views of A and C, respectively. Dots were drawn with 10% opacity.

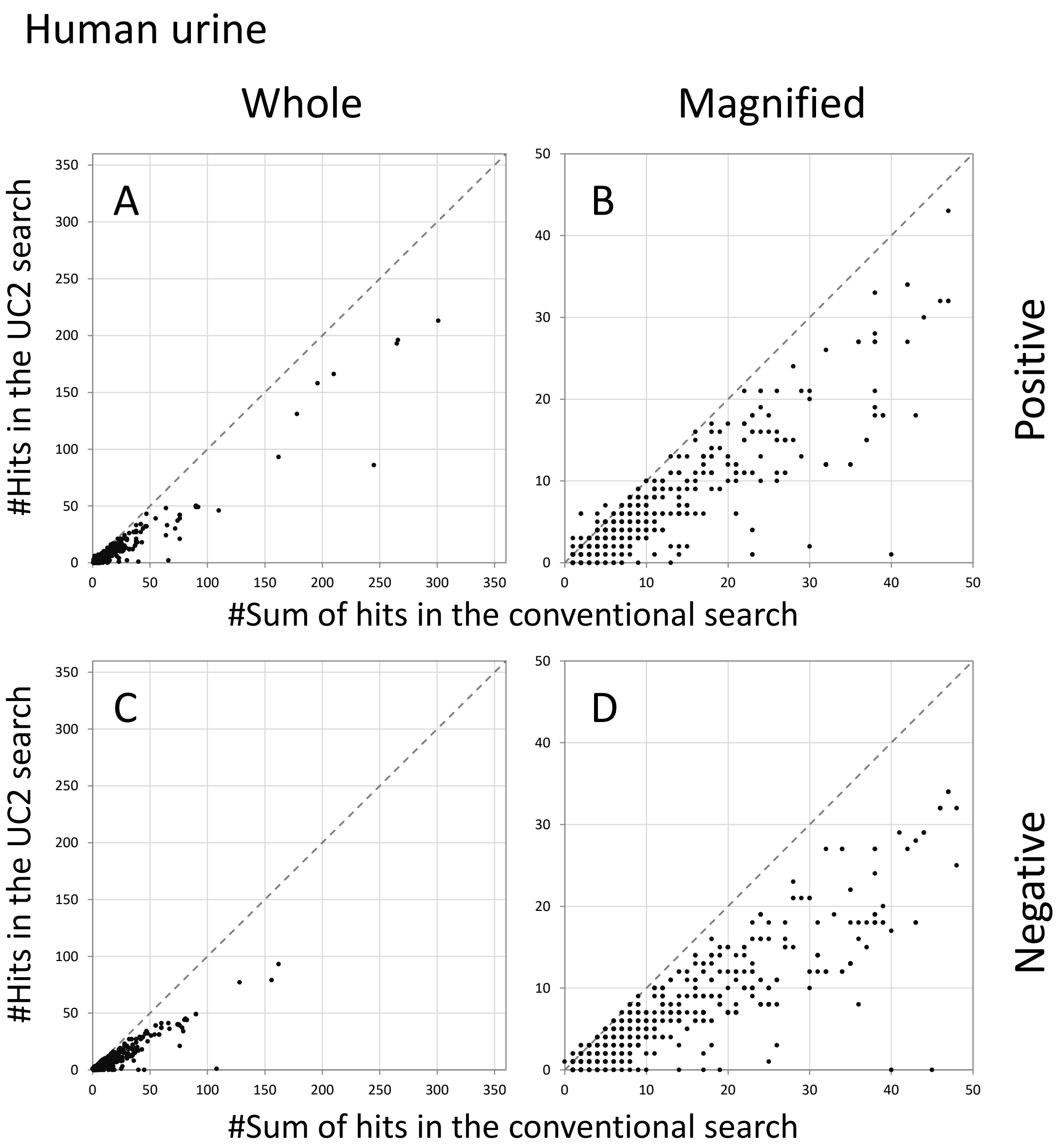

Supplementary Fig. S13. Comparison of the number of hits in the conventional search and the UC2 search for metabolites in human urine. The number of compound hits in the conventional search and in the UC2 search using the databases (KEGG, KNApSAcK, FlavonoidViewer, HMDB and LIPID MAPS) is plotted. The sum of search results with neutralized mass value and detected *m/z* value was used in the results of the conventional search. The mass values of the metabolite peaks detected in human urine in positive (A and B) and negative (C and D) modes were searched with 5 ppm mass tolerance. B and D are magnified views of A and C, respectively. Dots were drawn with 10% opacity.

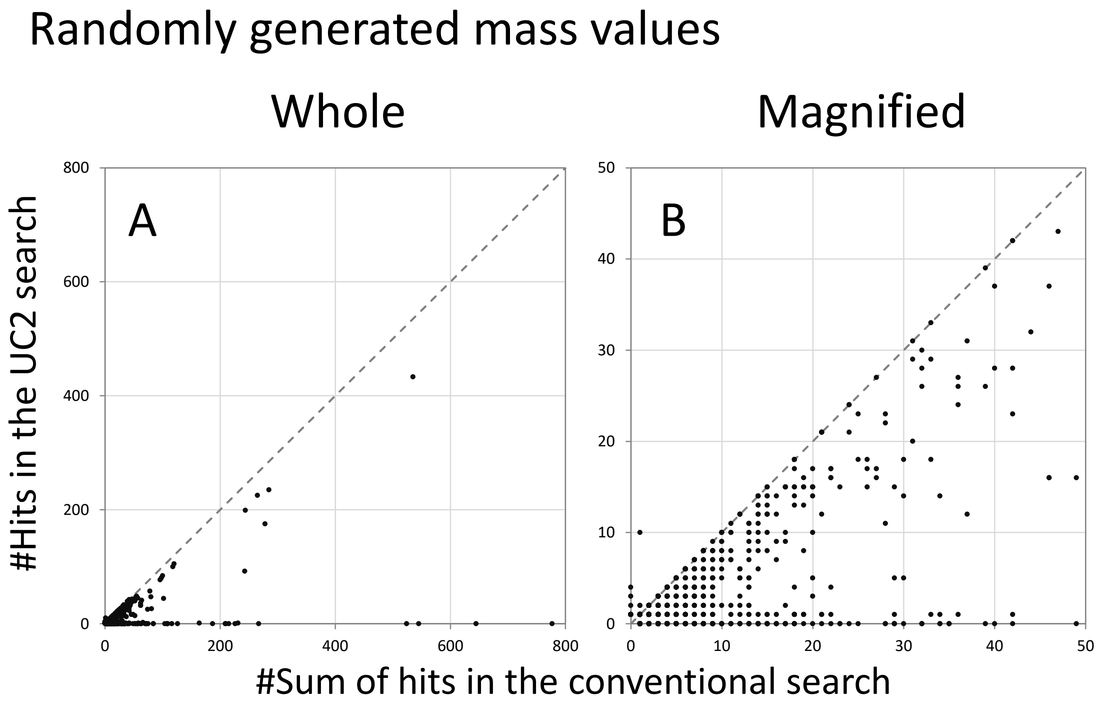

Supplementary Fig. S14. Comparison of the number of hits in the conventional search and the UC2 search for randomly generated mass values. The number of compound hits in the conventional search and in the UC2 search using the databases (KEGG, KNApSAcK, FlavonoidViewer, HMDB and LIPID MAPS) is plotted. A protonated cation ($[M+H]^+$) and a deprotonated anion ($[M-H]^-$) were assumed for neutralized mass values in the positive and negative modes, respectively. The sum of search results with neutralized mass value and generated mass value was used in the results of the conventional search. A mass tolerance of 5 ppm was allowed. B is a magnified view of A. Dots were drawn with 10% opacity.

References

Afendi, F.M. *et al.* (2012) KNApSAcK family databases: integrated metabolite-plant species databases for multifaceted plant research, *Plant Cell Physiol.*, **53**, e1.

de Laeter, J.R. *et al.* (2003) Atomic weights of the elements: Review 2000 (IUPAC Technical Report), *Pure Appl. Chem.*, **75**, 683-800.

Fahy, E. *et al.* (2009) Update of the LIPID MAPS comprehensive classification system for lipids, *J. Lipid Res.*, **50 Suppl**, S9-14.

Gu, J. *et al.* (2013) Use of natural products as chemical library for drug discovery and network pharmacology, *PLoS One*, **8**, e62839.

Heller, S. *et al.* (2013) InChI - the worldwide chemical structure identifier standard, *J. Cheminf.*, **5**, 1-9.

Iijima, Y. *et al.* (2008) Metabolite annotations based on the integration of mass spectral information, *Plant J.*, **54**, 949-962.

Kanehisa, M. *et al.* (2016) KEGG as a reference resource for gene and protein annotation, *Nucleic Acids Res.*, **44**, D457-462.

Kessner, D. *et al.* (2008) ProteoWizard: open source software for rapid proteomics tools development, *Bioinformatics*, **24**, 2534-2536.

Sakurai, N. *et al.* (2014) Tools and databases of the KOMICS web portal for preprocessing, mining, and dissemination of metabolomics data, *BioMed Res. Int.*, **2014**, 1-11.

Sakurai, N. *et al.* (2013) An application of a relational database system for high-throughput prediction of elemental compositions from accurate mass values, *Bioinformatics*, **29**, 290-291.

Salek, R.M. *et al.* (2013) The MetaboLights repository: curation challenges in metabolomics, *Database: the journal of biological databases and curation*, **2013**, bat029.

van der Hooft, J.J.J. *et al.* (2016) Urinary antihypertensive drug metabolite screening using molecular networking coupled to high-resolution mass spectrometry fragmentation, *Metabolomics*, **12**, 125.

Wang, Y. *et al.* (2009) PubChem: a public information system for analyzing bioactivities of small molecules, *Nucleic Acids Res.*, **37**, W623-633.

Willighagen, E.L. *et al.* (2017) The Chemistry Development Kit (CDK) v2.0: atom typing, depiction, molecular formulas, and substructure searching, *J. Cheminf.*, **9**.

Wishart, D.S. *et al.* (2013) HMDB 3.0—The Human Metabolome Database in 2013, *Nucleic Acids Res.*, **41**, D801-807.
